# Personality disorder services in England: findings from a national survey

**DOI:** 10.1192/pb.bp.116.055251

**Published:** 2017-10

**Authors:** Oliver Dale, Faisil Sethi, Clive Stanton, Sacha Evans, Kirsten Barnicot, Rosemary Sedgwick, Steve Goldsack, Monica Doran, Lucinda Shoolbred, Chiara Samele, Norman Urquia, Rex Haigh, Paul Moran

**Affiliations:** 1 West London Mental Health Trust, London, UK; 2 South London and Maudsley NHS Foundation Trust, London, UK; 3 University of New South Wales, Sydney, Australia; 4 Central and North West London NHS Foundation Trust, London, UK; 5 Imperial College London, UK; 6 Medway Engagement Group and Network (MEGAN) CIC, Chatham, UK; 7 Informed Thinking, London, UK; 8 Berkshire Healthcare NHS Foundation Trust, UK; 9 University of Bristol, Bristol, UK

## Abstract

**Aims and method** We aimed to evaluate the availability and nature of services for people affected by personality disorder in England by conducting a survey of English National Health Service (NHS) mental health trusts and independent organisations.

**Results** In England, 84% of organisations reported having at least one dedicated personality disorder service. This represents a fivefold increase compared with a 2002 survey. However, only 55% of organisations reported that patients had equal access across localities to these dedicated services. Dedicated services commonly had good levels of service use and carer involvement, and engagement in education, research and training. However, a wider multidisciplinary team and a greater number of biopsychosocial interventions were available through generic services.

**Clinical implications** There has been a substantial increase in service provision for people affected by personality disorder, but continued variability in the availability of services is apparent and it remains unclear whether quality of care has improved.

Personality disorder is a serious mental health condition affecting up to 52% of psychiatric out-patients and 70% of in-patients and forensic patients.^1–4^ Given the significant public health implications associated with the disorder – including extensive use of healthcare resources, high rates of suicide and reduced life expectancy – effective treatment is a priority.^5–8^

In 2003, the National Institute for Mental Health England (NIMHE) published *Personality Disorder: No Longer a Diagnosis of Exclusion*, challenging the healthcare community to address shortcomings in the treatment of people with personality disorders.^9^ Citing a survey of English mental health trusts conducted in 2002, the paper brought to attention the variability in practice and highlighted institutionalised stigmatisation which explicitly barred patients with personality disorder from mainstream services. At that time, only 17% of trusts had a dedicated personality disorder service, 40% provided some level of service, 28% had no identified service, and 25% did not respond.^9^

The 2003 NIMHE publication^9^ set out broad principles for how personality disorder services should be developed, stipulating that they should be multidisciplinary, follow a hub-and-spoke model, accept the management of risk, use the care programme approach (CPA),^10^ offer specialist biopsychosocial interventions, deliver training and consultation, and support the development of patient networks. Similarly, the 2009 guidance on borderline personality disorder from the National Institute for Health and Care Excellence (NICE) specified that mental health trusts should develop specialist multidisciplinary teams and/or services for people with personality disorders.^11,12^ In 2011, a preliminary investigation at a regional level found that specialist service capacity for those with personality disorder was inadequate.^13^ There have been no systematic attempts at a national scale aimed at understanding how the evidence for the management of personality disorder is being applied or whether service availability has become more uniform.

In 2014, the National Personality Disorder Service Review Group was formed to evaluate the extent to which variable service availability affects those with personality disorder. The group used the vision of *Personality Disorder: No Longer a Diagnosis of Exclusion* as its benchmark. Through this lens, we sought to map the availability and nature of dedicated personality disorder services, and to compare these to the care for clients with personality disorder available through generic services. The group drew on a wide range of evidence to define the concept of a ‘dedicated personality disorder service’. This included the Delphi study of the 11 pilot personality disorder projects within the National Personality Disorder Programme.^14^ We considered a dedicated service as one which is explicitly designed to manage the care of individuals affected by personality disorder, as opposed to a generic service which might be considered a typical community mental health service. Table 1 displays the characteristics hypothesised by the group to distinguish dedicated from generic services.

**Table 1:** Summary of service characteristics

Dedicated personality disorder services	Generic services
Personality disorder inclusion	No diagnostic inclusion/exclusion criteria

Variable service availability	Ubiquitous

Personality disorder-specific interventions	Offer range of biopsychosocial interventions

Specialist team	Mainstream multidisciplinary team

Local, regional and national catchment	Local catchment

Variable tiers (T1 to T6)	Locally focused tiers (T2 to T3)

Target complexity	Range of complexity

Treatment, consultation and training	Treatment orientation

Variable framework (includes CPA)	Operate under CPA framework

CPA, care programme approach.

## Aims

The primary aim of this study was to describe a number of organisations which provided care for those affected by personality disorder and whether this care was delivered through dedicated personality disorder services, generic services or both.

The secondary aim was to evaluate the provision of services for personality disorder along key quality indicators outlined by NICE and NIMHE,^9,11,12^ and explore any differences between dedicated and generic services. The quality indicators evaluated were:

1. Is there a multidisciplinary team available?
2. Is care managed under the CPA process?
3. Are patients offered specific interventions for personality disorder within a biopsychosocial approach?
4. Are services involved in education, training and research?
5. What level of patient and carer involvement do services employ?
6. What exclusion criteria, if any, are applied by services?

## Method

### Survey design

We conducted a cross-sectional survey of mental health organisations in England using a questionnaire designed for this study. Data were collected between January and June 2015 using an online survey tool (www.surveymonkey.com).

### Sample

The sample included any English mental health National Health Service (NHS) trust or independent provider of mental healthcare to adults or young people. In 2015 there were 57 relevant English mental health NHS trusts and 10 independent service providers, all of whom were approached to participate in the survey.

### Procedure

Letters were sent to the medical directors of each NHS mental health trust and the CEOs of the independent providers informing them of the survey and requesting the name of the individual who they considered to have the requisite knowledge to complete the survey. Once details of these individuals were obtained, letters were sent inviting them to take part. Non-responders were followed up at least twice where necessary, offering further information or support to complete the survey questionnaire.

### Survey questionnaire

Following an analysis of available literature, the electronic survey was structured to address the primary and secondary aims of the project. Participants were asked to briefly describe their organisation (e.g. NHS or independent provider, geographical remit) and their own professional role. They were then given a brief definition of a dedicated personality disorder service and of a generic service and asked to indicate whether their organisation had services of each type and detailed questions about its characteristics. We requested details of a maximum of five dedicated personality disorder services per organisation.

Questions relating to service characteristics included service leadership, team make-up, service access, inclusion and exclusion criteria, care management framework, intervention availability, patient and carer involvement, and training, education and research activity. The survey took up to 45 minutes to complete and could be conducted electronically or with telephone support.

### Data analysis

Data were downloaded from Survey Monkey and entered initially into Excel for checking and data cleaning, and transferred to STATA (version 11) for statistical analysis. To address the primary aim of the survey, the characteristics of services were summarised descriptively in order to build a picture of service availability and characteristics. The availability of biopsychosocial interventions was assessed by generating a score ranging from 0 to 100 based on the number of available interventions of each type, weighted to give equal consideration to each of the three domains. The availability of personality disorder-specific interventions was assessed by determining whether services offered psychological therapies developed specifically for personality disorder.^15^ The level of perceived patient and carer involvement was similarly analysed and scored from 0 to 100 based on the number of involvement activities for each service, with paid involvement double weighted.

To address the secondary aims of the survey, logistic and linear regression was used to evaluate the effect of service type (dedicated or generic) on professional diversity, exclusion criteria, CPA usage, biopsychosocial provision, patient and carer involvement, and training, education and research activity. Multilevel models, with a random effect for organisation, were used to adjust for the potential higher similarity between services within the same organisation than between services from different organisations. Robust standard errors were used for linear variables that did not conform to a normal distribution. Where significant differences between dedicated and generic services were found, multivariate models were used to adjust for the influence of potentially confounding service characteristics.

## Results

### Respondents

Of the 57 relevant English mental health NHS trusts, 52 responded (response rate 91%) and of the 10 independent service providers approached 4 responded (response rate 40%).

### Primary study aim: availability of services for people with personality disorder

Of the 56 organisations that responded to the survey, 47 (84%) reported having at least one dedicated personality disorder service and 43 (77%) reported having both generic and dedicated services. The remaining 4 organisations (7%) stated that they did not have any generic services and that all services were specialist; all offered dedicated personality disorder services. Nine organisations (16%) did not have any dedicated personality disorder services, and all of these stated that their generic services catered to personality disorder. Patients were reported to have equal access to dedicated personality disorder services in 31 (55%) of the organisations surveyed.

**Fig. 1:**
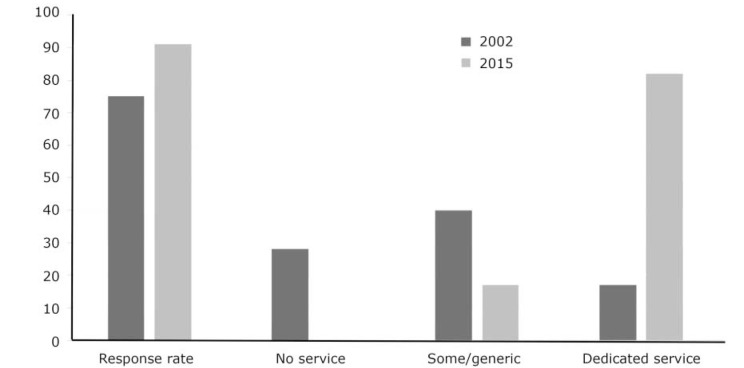
Comparison between 2002 and 2015 survey results (percentage change; English mental health NHS trusts only).

The number of dedicated personality disorder services per organisation ranged from 1 to 5 (mean 1.7, s.d. = 1.1). Across the 52 English mental health NHS trusts, 71 dedicated personality disorder services and 48 generic services were described, a mean of 1.37 dedicated service per organisation (range 0–5). The four independent service providers described ten dedicated personality disorder services; a mean of 2.50 dedicated service per organisation (range 1–5). Figure 1 compares the findings with the survey of 2002. To aid comparison, the independent sector organisations have been removed from the 2015 results so that only English NHS mental health trusts are referred to. Tables 2, 3, 4, 5 and 6 summarise the characteristics of the dedicated and generic services across all domains surveyed.

**Table 2:** Summary of service and team characteristics

	Dedicated *n* (%)	Generic *n* (%)
Tier 1	9 (11)	

Tier 2	43 (53)	

Tier 3	45 (56)	

Tier 4	17 (21)	

Tier 5	11 (14)	

Tier 6	1 (1)	

Service level leadership		
Consultant clinical psychologist	26 (32)	6 (12)
Consultant medical psychotherapist	21 (26)	2 (4)
Consultant psychiatrist	13 (16)	25 (49)
Consultant nurse	8 (10)	0 (0)
Clinical psychologist	4 (5)	1 (2)
Consultant adult psychotherapist	3 (4)	0 (0)
Non-medical psychotherapist	1 (1)	0 (0)
Undisclosed	3 (4)	16 (31)
Other	2 (2)	2 (4)

Team constitution		
Nurse	56 (69)	45 (88)
Consultant clinical psychologist	41 (51)	29 (57)
Trainee psychologist	37 (46)	40 (78)
Consultant medical psychotherapist	36 (44)	18 (35)
Clinical psychologist	32 (40)	44 (86)
Occupational therapist	32 (40)	42 (82)
Social worker	31 (38)	38 (75)
Non-medical psychotherapist	30 (37)	25 (49)
Peer worker	26 (32)	26 (51)
Consultant adult psychiatrist	25 (31)	45 (88)
Trainee psychiatrist	24 (30)	37 (73)
Consultant nurse	20 (25)	21 (41)
Advocate	13 (16)	16 (31)
Consultant forensic psychiatrist	10 (12)	14 (27)
Pharmacist	10 (12)	25 (49)
Forensic psychologist	6 (7)	14 (27)
Trainee medical psychotherapist	4 (5)	13 (25)
Probation professional	4 (5)	5 (10)
Consultant forensic psychologist	3 (4)	11 (22)
Dual diagnosis professional	2 (2)	17 (33)

Clinical management framework		
Under CPA	64 (79)	47 (92)
Not under CPA	9 (11)	2 (4)
Not applicable	8 (10)	6 (12)

CPA, care programme approach.

**Table 3:** Summary of interventions offered

	Dedicated service	Generic service
Biological interventions, *n* (%)		
Medication management	42 (52)	46 (90)
Organic investigations	28 (35)	42 (82)
Physical healthcare interventions	27 (33)	44 (86)
MUS management	23 (28)	27 (53)

Psychological interventions, *n* (%)		
Psychoeducation	44 (54)	40 (78)
DBT	40 (49)	29 (57)
MBT	35 (43)	21 (41)
Psychodynamic	30 (37)	27 (53)
CBT	27 (33)	42 (82)
CAT	26 (32)	35 (69)
Art therapies	22 (27)	25 (49)
Therapeutic community	19 (23)	9 (18)
Family therapy	14 (17)	26 (51)
Motivational interviewing	14 (17)	22 (43)
SFT	10 (12)	23 (45)
STEPPS	8 (10)	8 (16)

Social interventions, *n* (%)		
Peer support	39 (48)	26 (51)
Vocational support	37 (46)	34 (67)
Occupational therapy	35 (43)	41 (80)
Social work	32 (40)	36 (71)
Housing support	31 (38)	37 (73)
Benefits advisory	28 (35)	28 (55)
Advocacy	25 (31)	31 (61)

Bio-psychosocial interventions rating, mean (s.d.)	3.4 (2.5)	6.3 (2.0)

CAT, cognitive–analytic therapy; CBT, cognitive–behavioural therapy; DBT, dialectical behaviour therapy; MBT, mentalisation-based therapy; MUS, medically unexplained symptoms; SFT, schema-focused therapy; STEPPS, systems training for emotional predictability and problem solving.

**Table 4:** Summary of development activities

Integrated development activities	Dedicated *n* (%)	Generic *n* (%)
Training	75 (93)	27 (53)

Education	66 (81)	22 (43)

Research	56 (69)	18 (35)

Training + education + research	48 (59)	13 (25)

**Table 5:** Summary of patient and carer involvement

	Paid, *n* (%)		Voluntary, *n* (%)	
	Dedicated	Generic	Dedicated	Generic
Patient				
Service development	25 (31)	24 (47)	33 (41)	23 (45)
Education and training	25 (31)	19 (37)	26 (32)	20 (39)
Treatment	14 (17)	11 (19)	16 (20)	13 (25)
Service delivery	14 (17)	5 (10)	20 (25)	16 (31)
Leadership	11 (14)	11 (22)	14 (17)	10 (20)
Care planning	7 (9)	8 (16)	19 (23)	13 (25)
None	18 (22)	19 (37)	9 (11)	20 (39)

Carer				
Service development	1 (1)	1 (2)	11 (14)	11 (22)
Education and training	3 (4)	4 (8)	5 (6)	7 (14)
Service delivery	2 (2)	3 (6)	3 (4)	7 (14)
Care planning	0 (0)	3 (6)	10 (12)	9 (18)
Treatment	0 (0)	2 (4)	2 (2)	5 (10)
Leadership	3 (4)	2 (4)	2 (2)	3 (6)
None	14 (17)	39 (76)	9 (11)	32 (63)

	Dedicated		Generic	
Patient and carer involvement rating, mean (s.d.)	12.4 (12.3)		6.3 (5.6)	

**Table 6:** Service level exclusion

Criteria	Dedicated *n* (%)	Generic *n* (%)
Uncontrolled substance misuse	43 (53)	10 (20)

Active risk to others	19 (23)	2 (4)

Ability to engage	16 (20)	4 (8)

Comorbid psychotic disorder	14 (17)	1 (2)

Developmental disorder	12 (15)	1 (2)

Gender	11 (14)	1 (2)

Forensic history	6 (7)	1 (2)

Comorbid affective disorder	6 (7)	1 (2)

Active risk to self	4 (5)	2 (4)

Past risk to others	2 (2)	0 (0)

Past risk to self	0 (0)	0 (0)

Prescribed medication	0 (0)	0 (0)

None	18 (22)	35 (69)

Others or not applicable	7 (9)	6 (12)

### Secondary study aims: quality indicators of available services

#### 1. Is there a multidisciplinary team available?

Across services, teams varied widely in their multi-disciplinary composition (Table 2). Within services, team make-up was significantly less diverse in dedicated than in generic services, with the latter utilising almost twice as many different types of professional on average (dedicated personality disorder services mean 5.7, s.d. = 3.0; generic services mean 10.5, s.d. = 5.1 (β = −4.85, 95% CI −6.37 to −3.32, *P* < 0.01)). Dedicated services remained less professionally diverse than generic services after adjusting for the range of biopsychosocial interventions available, the provision of personality disorder-specific interventions, and the profession of the service lead (β = −3.14, 95% CI −4.46 to −1.82, *P* < 0.01). This suggests that the less diverse workforce in dedicated services was not simply due to providing a more focused range of interventions.

#### 2. Is care managed under the CPA process?

Almost all services used the CPA as their management framework. There was no difference between dedicated and generic services in CPA usage (odds ratio (OR) = 0.22, 95% CI 0.04 to 1.47, *P* = 0.12).

#### 3. Are patients offered specific interventions for personality disorder within a biopsychosocial approach?

Across services, there was a fairly wide availability of a number of different biological, psychological and social interventions. Table 3 includes the mean biopsychosocial ratings stratified by service type. Generic services had significantly higher biopsychosocial ratings than dedicated ones, indicating a greater availability and diversity of interventions (β = 3.02, 95% CI 2.32 to 3.73, *P* < 0.01). However, services led by medics offered a greater range of interventions than those led by other professionals (β = 1.09, 95% CI 0.97 to 2.84, *P* < 0.01), as did services with a more diverse professional make-up (β = 0.38, 95% CI 0.32 to 0.45, *P* < 0.01). Biopsychosocial intervention provision did not differ between dedicated and generic services after adjusting for these factors (β = 0.69, 95% CI −0.29 to 1.68, *P* = 0.17). Contrary to hypothesis, the availability of interventions developed specifically for personality disorder (such as dialectical behaviour therapy (DBT), mentalisation-based therapy (MBT), schema-focused therapy (SFT) and systems training for emotional predictability and problem solving (STEPPS)) did not differ significantly between dedicated and generic services (OR = 0.91, 95% CI 0.37 to 2.21, *P* = 0.83).

#### 4. Are services involved in education, training and research?

Most services were involved in at least one of these activities (Table 4). The rates of participation in these activities for dedicated services were approximately twice those of generic services, and dedicated services were significantly more likely than generic services to be involved in all three of these activities (i.e. education, training and research) (OR = 6.18, 95% CI 2.29 to 16.69, *P* < 0.01). This difference remained significant after adjusting for the profession of the service lead and for the professional diversity of the team (OR = 31.67, 95% CI 4.26 to 235.5, *P* < 0.01).

#### 5. What level of patient and carer involvement do services employ?

Table 5 contains the mean patient and carer ratings stratified by service type. Very few services had no patient or carer involvement, and the odds of having any involvement activity did not differ between dedicated and generic services (OR = 1.17, 95% CI 0.42 to 3.22, *P* = 0.77). However, dedicated services had significantly higher patient and carer involvement ratings than generic ones, indicating involvement in a greater number of service development, care planning, service delivery, training and leadership activities (β = 6.29, 95% CI 3.03 to 9.55, *P* < 0.01). This difference remained significant after adjusting for the profession of the service lead and for the professional diversity of the team (β = 9.76, 95% CI 3.90 to 15.62, *P* < 0.01).

#### 6. What exclusion criteria, if any, are applied by services?

No services excluded individuals on the basis of a diagnosis of personality disorder. Across both dedicated and generic services, the most common exclusion criterion was uncontrolled substance misuse, followed by active risk to others (Table 6). Almost half of services (43%) had no exclusion criteria. Dedicated services were significantly more likely than generic ones to have exclusion criteria (OR = 10.95, 95% CI 3.31 to 36.19, *P* < 0.01). This difference remained significant after adjusting for the profession of the service lead and for the professional diversity of the team (OR = 5.02, 95% CI 1.24 to 20.35, *P* = 0.02).

## Discussion

This national survey was the first of its kind and captured data provided by 56 relevant mental health organisations in England. With a response rate of 91% for English mental health NHS trusts, and a sample of independent service providers, we can be confident the survey is representative of personality disorder provision in England.

The majority of organisations described both dedicated personality disorder services (84%) and generic services (91%), and in organisations with no dedicated services all provision for personality disorder was through a generic service. This quantifies the progress made in this area since 2002 and points to a fivefold increase in organisations providing dedicated personality disorder services.^9^

This represents substantial progress in a decade in which the economic landscape has been challenging. Yet, while on this measure we can see substantial progress at an organisational level, the survey indicates a worrying level of variability at a local level, with only 55% (*n* = 31) of organisations indicating equal access to the dedicated services they provide.

The 2003 NIMHE publication formally introduced the concept of dedicated personality disorder service as distinguished from generic service, and this distinction has been further developed in the current paper.^9^ We had *a priori* assumptions about the nature of dedicated and generic services (Table 1), and this survey allows a more detailed conceptual analysis. The survey methodology steered respondents to consider the concept of dedicated *v*. generic services. Analysis of the descriptive and statistical differences between the 81 dedicated and 51 generic services allows us to draw some conclusions about these two types of service provision. For instance, we found that generic services draw from a wide range of professional disciplines, which is in line with their broader remit. Seemingly, dedicated services draw from a more restricted range of professional disciplines; this supports the notion that they are specialist, niche services.

Contrary to our *a priori* hypothesis, dedicated services were no more likely to provide personality disorder-specific interventions when compared with generic services. Furthermore, generic services provide a significantly wider range of biopsychosocial interventions than dedicated ones, although there was some suggestion that this was influenced by their employment of a significantly more diverse workforce and by their higher rates of medical professional leadership. The accessibility of these interventions and the quality of their delivery are unknown; however, NICE guidance stipulates that specialist interventions are best delivered by specialist services.^11^

The delivery of developmental activities is a clear priority for dedicated services, with almost all involved in training, and significantly more dedicated than generic services involved, indicating that they deliver both training and education and research. This is in keeping with both the 2003 NIMHE publication and NICE guidance.^9,11,12^ Patient and carer involvement is also prioritised by dedicated services, with patients and carers involved in significantly more service development, management and delivery activities than those in generic services. Dedicated services appear to show greater selectivity in patient choice than generic ones, as significantly more operate with exclusion criteria. Given that impulsivity is a diagnostic criterion for borderline and dissocial personality disorder, it is noteworthy that active risk to others (23%) and substance misuse (53%) were so widely quoted as exclusion criteria for dedicated personality disorder services.

### Limitations

The response rate for the independent providers should be treated with caution as it is subject to selection bias. Responses were self-reported and there may have been variation in the interpretation of what constituted a dedicated personality disorder service.

In the comparisons made with generic services, the respondents were asked to provide an overview of all of the generic services within their organisation. Although this was pragmatically necessary, given the large numbers of generic services within any organisation, this approach requires the reader to consider the comparisons with appropriate caution. In particular, the findings which relate to the personality disorder-specific interventions and range of staff within the multidisciplinary team will be skewed by this methodology.

While this survey is able to give a good organisational-level description of service availability, mapping the local provision is achieved to a limited degree. Perhaps the most important consideration is that the indicators used in this survey to consider the quality can only provide a broad brush-stroke indication, owing to necessary methodological trade-offs for pragmatic purposes.

Understanding the consistency with which individual patients and carers can expect adherence to best practice and the timeliness of the interventions offered is beyond the scope of this survey. We believe this body of work begins to elucidate the questions which need to be considered, but it is a long way from achieving that. Indeed, the largest limitation of this work is that at best it provides a broad overview of provision. To properly understand what is actually delivered to those in need will require a more systematic and sustained effort to describe quality standards and ensure, perhaps through accreditation, that best practice is being followed.

### Further developments

This paper charts the most systematic attempt to date at mapping the provision of care across England for those affected by personality disorder. What is clear is that the past decade or so has seen considerable progress in providing a service for this range of disorders. Despite this progress, data presented here provide evidence that there remains continued exclusion, variability of practice and inconsistencies in the availability of services.

The current NICE guidance, in step with the evidence base, supports the provision of a range of cost-effective interventions and the establishment of specialist services from which to deliver them. The initial offering presented here lends weight to the call for the establishment of authoritative commissioning guidance and service standards to ensure that patients and carers have access to the care that they need.
